# Choline Oxidase-Incorporated ATRP-Based Cerium Nanogels as Nanozymes for Colorimetric Detection of Hydrogen Peroxide and Choline

**DOI:** 10.3390/bios14120563

**Published:** 2024-11-21

**Authors:** Trung Hieu Vu, Byung Jo Yu, Moon Il Kim

**Affiliations:** 1 Department of BioNano Technology, Gachon University, 1342 Seongnamdae-ro, Sujeong-gu, Seongnam 13120, Republic of Korea; hieu.vutrung24596@gmail.com; 2 Low-Carbon Transition R&D Department, Research Institute of Sustainable Development Technology, Korea Institute of Industrial Technology (KITECH), Cheonan 31056, Republic of Korea

**Keywords:** ATRP-based nanogels, cascade reaction, peroxidase-like nanozymes, choline detection, food safety

## Abstract

Choline is an important molecule in monitoring food safety and infant nutrition. Here, we report Ce nanogels synthesized by atom transfer radical polymerization (ATRP) employing Ce-coordinated acryloyl-lysine polymer brushes (Ce@SiO_2_ NGs) as highly efficient cascade nanozymes for colorimetric detection of choline. The synthesized Ce@SiO_2_ NGs demonstrated remarkable peroxidase-like activity with a porous exterior, which are essential to entrap choline oxidase (COx) to yield COx@Ce@SiO_2_ NGs and construct a cascade reaction system to detect choline. Immobilized COx catalyzed the oxidation of choline in food samples to produce H_2_O_2_, which subsequently induced the oxidation of chromogenic substrate 3,3′,5,5′-tetramethylbenzidine (TMB) to produce blue color signals. This method enabled the selective and sensitive detection of target choline with a satisfactory linear range of 4–400 μM, which is sufficient to analyze foodborne choline. The practical utility of the COx@Ce@SiO_2_ NG-based assay was successfully validated to determine choline spiked in commercially available milk and infant formula with high accuracy and precision values. This approach provides a simple and affordable method of choline detection and has the potential to lead to more developments in ATRP-based nanozymes for diverse biosensing applications.

## 1. Introduction

Choline is an essential nutrient for developing the brain and maintaining overall health [1,2,3]. Several diseases, such as Alzheimer’s disease, liver cirrhosis, and growth disorder, were suggested to be induced by long-term choline deficiency [4,5]. Usually, the body can restore choline through food intake, and, thus, accurate determination of choline in foods is an important issue. The US Food and Drug Administration (FDA) as well as the food and nutrition board (FNB) of the institute of medicine thus set regulations that infant formula not made from cow’s milk must be supplemented with a high choline concentration (up to 200 mg/100 g sample), which is equivalent to that of breast milk (~1 mM) [6]. In addition, H_2_O_2_ is commonly used as a disinfectant in food preservation; however, its excessive levels can pose health risks, such as oxidative stress and tissue damage [7,8]. Therefore, ensuring accurate quantification of choline as well as H_2_O_2_ in foods is essential for maintaining health standards and meeting dietary needs [6,9]. The traditional detection methods for choline and H_2_O_2_, such as high-performance liquid chromatography (HPLC) and fluorescence-based techniques, are generally selective and sensitive. However, these approaches present several challenges, including time-consuming sample pre-/post-treatment, interference from background signals, and the inevitable use of complicated instrumentation [7,10,11]. Thus, the development of more convenient, reliable, and sensitive methods for detecting choline and residual H_2_O_2_ is vital to overcome these limitations and improve food safety and nutritional assessments.

Recent advancements in nanotechnology have highlighted the potential of nanomaterials with enzyme-like properties, known as nanozymes, to replace natural enzymes in versatile applications. Nanozymes possess several superiorities to natural enzymes, such as enhanced stability and durability, tunable activity, and more affordable synthesis [12,13]. Despite these advantages, natural enzymes often outperform nanozymes in terms of substrate specificity and catalytic efficiency. This is primarily due to the intricate and finely tuned structure of the active sites in natural enzymes, which nanozymes struggle to replicate. To bridge this gap, recent studies have focused on designing more sophisticated nanozyme architectures, with attention on engineering their active sites. Notably, materials with metal–nitrogen (M–N) active sites, such as Fe-N, Co-N, Zn-N, or metal–organic frameworks (MOFs), have emerged as highly promising candidates [14,15,16,17]. M–N active site materials have mimicked the active centers of natural metalloenzymes, while MOFs provide nanoscale cavities resembling the three-dimensional (3D) binding pockets of natural enzymes, creating a conducible environment for the catalytic process. These advances in nanozyme development provide new opportunities in versatile applications from biochemical sensing and environmental remediation to medical theranostics [18].

Building on these developments, nanogels synthesized via ATRP have emerged as a promising platform for improving the catalytic performance of nanozymes. ATRP is recognized as a controlled polymerization method that enables the precise synthesis of well-defined, biocompatible nanogels. These nanogels are characterized by exceptional biocompatibility, tunable porosity, and the ability to create dynamic microenvironments around catalytic centers that resemble the active sites of natural enzymes [19,20,21,22,23]. Recently, an enzyme-catalyzed variant of this method, called ATRPase, was introduced. This approach enables the synthesis of biocompatible polymer brushes through the interfacial polymerization of amino-acid-based monomers, such as N-acryloyl-l-lysine. By coordinating with certain metal ions including Fe, these polymeric nanogels exhibited unique enzyme-like activities [24,25]. Although the potential of ATRP-based nanozymes has been demonstrated, further investigations are required to study the effects of versatile metal ions regarding incorporation within the nanogels to induce affirmative enzyme-like activity for their practical applications.

Cerium (Ce) ions exhibit exceptional qualities as effective cross-linkers in the formation of various hydrogel-based materials. Their integration into the hydrogel matrix through cross-linking enhances the structural stability and mechanical properties of the hydrogels while enabling the creation of materials with tailored functionalities [26,27,28]. By leveraging the unique coordination chemistry of Ce ions, researchers have designed hydrogels with affirmative properties, such as improved stability, increased loading capacities, and enhanced catalytic activities, making them suitable for diverse applications in the biomedical and environmental fields [29,30]. Moreover, the ability of Ce ions to alternate between Ce^3+^ and Ce^4+^ oxidation states promotes strong peroxidase-like activity, making them highly effective for various biosensing applications [14]. Additionally, the monoatomic dispersion of Ce ions within the hydrophilic network of the nanogels possibly creates a high density of active sites, resulting in significantly improved reaction rates and overall catalytic efficiency [31,32]. These advantageous properties position Ce-based nanogels as promising materials for advanced catalytic and biosensing platforms.

Herein, we developed Ce nanogels synthesized by ATRP employing Ce-coordinated acryloyl-lysine polymer brushes (Ce@SiO_2_ NGs) as an efficient peroxidase mimic and scaffold for oxidative enzyme entrapment, which is designed for colorimetric detection of H_2_O_2_ and choline by incorporating COx within the nanogels. The Ce ions within the Ce@SiO_2_ NGs serve as both structural cross-linkers and active centers, leading to enhanced catalytic efficiency. Additionally, the ATRP-based nanogels provided a biocompatible and porous network that is capable of entrapping COx, resulting in COx@Ce@SiO_2_ NGs performing selective and sensitive choline detection via a cascade reaction. In the presence of choline, COx catalyzed its oxidation to produce H_2_O_2_, which subsequently activated the peroxidase-mimicking Ce@SiO_2_ NGs to oxidize a chromogenic substrate TMB to produce a blue color. This provides a dual-function platform capable of detecting choline as well as H_2_O_2_. By leveraging the catalytic properties of Ce@SiO_2_ NGs and COx@Ce@SiO_2_ NGs, a versatile, reliable, and scalable method has been developed to enhance food safety analysis, particularly in the context of dairy products and infant nutrition.

## 2. Materials and Methods

### 2.1. Reagents and Materials

2-Bromoisobutanoic acid N-hydroxysuccinimide ester (NHS-Bib), sodium hydroxide (NaOH), sodium carbonate (Na_2_CO_3_), sodium acetate (CH_3_COONa), sodium ascorbate, copper sulfate pentahydrate (CuSO_4_.5H_2_O), TMB, ammonia solution (28% in water), dimethyl sulfoxide (DMSO), tetraethyl orthosilicate (TEOS), cerium(III) nitrate hexahydrate (Ce(NO_3_)_3_.6H_2_O), absolute ethanol, acryloyl chloride, L-lysine hydrochloride, horseradish peroxidase (HRP), 3-aminopropyl triethoxysilane (APTES), phosphate buffered saline (PBS), and 8-hydroxyquinoline were purchased from Sigma-Aldrich (St. Louis, MO, USA). Hydrogen peroxide was obtained from Samchun Chemical (Seoul, Republic of Korea). All solutions were prepared with deionized (DI) water purified by a Milli-Q Purification System (Millipore, Darmstadt, Germany).

### 2.2. Material Characterizations

Using a Field Emission Scanning Electron Microscope (JSM-7500F JEOL, Pleasanton, CA, USA) for scanning electron microscopy (SEM) and a Transmission Electron Microscope (FEI Tecnai, Hillsboro, OR, USA) for transmission electron microscopy (TEM) and high-resolution TEM (HR-TEM), the morphology of the Ce@SiO_2_ NGs was examined. Energy-dispersive spectroscopy (EDS) was used to analyze the elemental composition (Bruker, Billerica, MA, USA). The suspension of sonicated Ce@SiO_2_ NGs was allowed to dry overnight on a silicon wafer in preparation for the SEM examinations. Further, 5 μL of the sonicated nanogel suspension was deposited onto a carbon-coated copper TEM grid (Electron Microscopy Sciences, Hatfield, PA, USA) for the TEM studies, and it was then allowed to dry overnight at room temperature (RT). An FT-IR spectrophotometer (FT/IR-4600, JASCO, Easton, MD, USA) was used to acquire the Fourier transform infrared (FT-IR) spectra of Ce@SiO_2_ NGs. X-ray photoelectron spectroscopy (XPS) was conducted using an XPS reader (Sigma Probe, Thermo Scientific, Madison, WI, USA) to analyze the contribution of elements. Water contact angle was measured to investigate the hydrophilicity of Ce@SiO_2_ NGs using a Phoenix 300 contact angle analyzer (Surface Electro Optics, Suwon, Gyeonggi, Republic of Korea).

### 2.3. Synthesis of Ce@SiO_2_ NGs and Ce@SiO_2_ NGs Entrapping COx (COx@Ce@SiO_2_ NGs)

Ce@SiO_2_ NGs were synthesized from SiO_2_ nanoparticles (NPs), N-acryloyl-L-lysine brush, and Ce(NO_3_)_2_ solution following reported method with minor modifications [24]. First, N-acryloyl-L-lysine solution (50 mg/mL), SiO_2_–Br NP dispersion (5 mg/mL), and sodium ascorbate solution (1.5 mg/mL) were mixed thoroughly in PBS (10 mM, pH 6) under nitrogen atmosphere. Next, HRP (5 mg/mL) as an ATRP catalyst (ATRPase) was added and the solution was oscillated overnight at RT. In order to obtain the nanogel layer at the surface of nanoparticles, Ce(NO_3_)_3_·6H_2_O was added to the solution and stirred for 2 h at RT to induce the cross-linking process of lysine moieties at the surface of polymer brushes (N-acryloyl-L-lysine). The Ce-coordinated nanogel on SiO_2_ surfaces (Ce@SiO_2_ NGs) was obtained by centrifugation at 5000× *g* for 5 min and washing with H_2_O and absolute ethanol. SiO_2_-Pol(Lys) NPs were also synthesized via the same method except the incorporation of Ce.

COx@Ce@SiO_2_ NGs were prepared by mixing Ce@SiO_2_ NGs with several concentrations of COx (0.025, 0.05, 0.1, 0.2, and 0.4 mg/mL) for 30 min at RT. The samples were collected by centrifugation at 5000× *g* for 5 min, followed by washing with DI water to obtain COx@Ce@SiO_2_ NGs. The concentrations of COx before and after the immobilization remaining in the supernatant were measured using the bicinchoninic acid (BCA) assay to calculate the loading capacity.

### 2.4. Evaluation of Peroxidase-like Activity of Ce@SiO_2_ NGs

Peroxidase-like activity of Ce@SiO_2_ NGs was assessed by monitoring the oxidation of TMB in the presence of H_2_O_2_. In this assay, Ce@SiO_2_ NGs (0.1 mg/mL) were added into a reaction buffer (0.1 M sodium acetate (NaAc), pH 4.0) containing TMB (1 mM) and H_2_O_2_ (10 mM) and incubated for 5 min at RT. Following the incubation, the catalytic materials were separated by centrifugation (13,000× *g*, 2 min). The absorbance of the supernatant was measured in a scanning mode from 550 to 750 nm or at 652 nm using a microplate reader (Synergy H1, BioTek, Winooski, VT, USA).

Stability of Ce@SiO_2_ NGs was compared with that of HRP by evaluating their activity in NaAc buffer (0.1 M) across varying conditions of temperature (4 to 90 °C) and pH (3 to 9). After incubation for 2 h, the residual activities of Ce@SiO_2_ NGs and HRP were measured using aforementioned colorimetric assays.

Steady-state kinetic studies were conducted to evaluate the kinetic parameters of Ce@SiO₂ NGs. The experiments were performed in NaAc buffer (0.1 M, pH 4.0) containing 0.1 mg/mL Ce@SiO_2_ NGs. For TMB, the reaction buffer was supplemented with 10 mM H_2_O_2_ at varying concentrations of TMB, while, for H_2_O_2_, 1 mM TMB was added in reaction buffer at various concentrations of H_2_O_2_. After addition of TMB or H_2_O_2_, absorbance was monitored by measuring the color changes of reaction solution using a kinetic mode at 652 nm. The kinetic parameters were calculated using the equation *v* = *V_max_* × [S]/(*K_m_* + [S]), where *v* is the initial velocity, *V_max_* is maximal velocity, [S] is substrate concentration, and *K_m_* is Michaelis constant.

### 2.5. Quantitative Determination of H_2_O_2_ Using Ce@SiO_2_ NGs

H_2_O_2_ concentration was determined using TMB as a substrate in a transparent 96-well plate as follows. Various concentrations of H_2_O_2_ were added in NaAc buffer (0.1 M, pH 4.0) containing Ce@SiO_2_ NGs (0.1 mg/mL) and TMB (1 mM). After incubation for 5 min at RT, the Ce@SiO_2_ NGs were separated and the absorbance of supernatant was measured at 652 nm using a microplate reader.

### 2.6. Quantitative Determination of Choline Using COx@Ce@SiO_2_ NGs

The quantification of choline level was conducted by incubating COx@Ce@SiO_2_ NGs with varying concentrations of choline in HEPES buffer (0.05 M, pH 7.0) at RT for 15 min. Then, NaAc buffer (0.1 M, pH 4) containing 1 mM TMB was added and incubated for 5 min. Following the incubation, further procedures were the same as those described for H_2_O_2_ detection.

### 2.7. Detection of H_2_O_2_ and Choline in Milk and Infant Formula Samples

Fresh milk and infant formula samples were purchased from local market and pretreated with methanol to remove organic impurities before being diluted 100 times with HEPES buffer. The amounts of H_2_O_2_ and choline in diluted samples were measured by HRP-TMB-based assay and choline assay kit (Abcam, Cambridge, UK), respectively. Then the prescribed amounts of H_2_O_2_ (25, 50, and 100 µM) and choline (50, 100, and 200 µM) were added into diluted samples to create spiked samples. The levels of choline and H_2_O_2_ in these spiked samples were subsequently analyzed using the same detection protocols as described earlier for individual H_2_O_2_ and choline quantification. To evaluate the accuracy and reproducibility of the assay, recovery rate (%) and coefficient of variation (CV, %) were calculated based on three independent assay results for each spiked sample. Recovery rate (%) and CV (%) are defined by these equations: [recovery (%) = measured value/actual value × 100] and [CV (%) = SD/average × 100].

## 3. Results and Discussion

### 3.1. Synthesis and Characterization of Ce@SiO_2_ NGs

**Figure 1:**
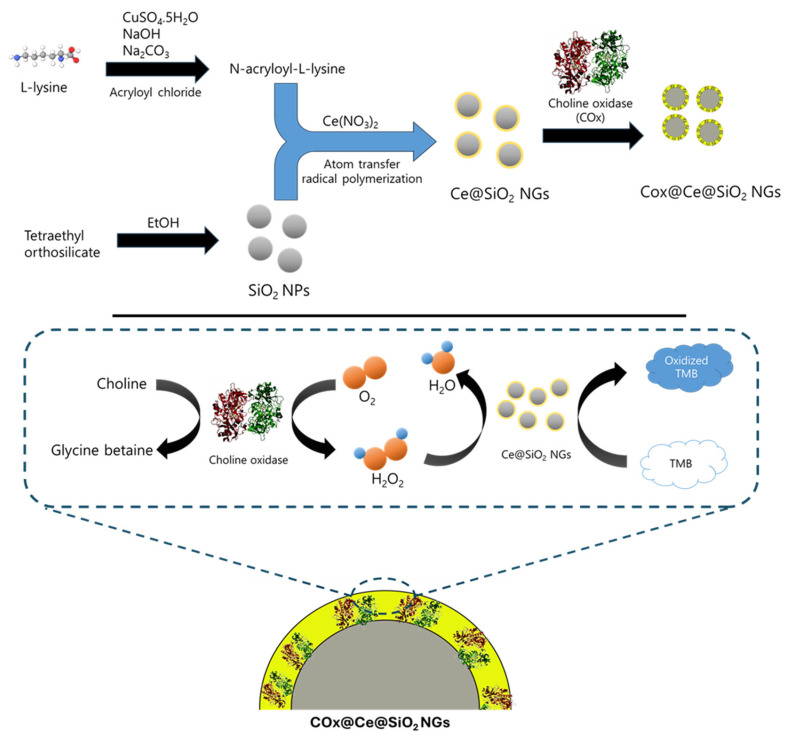
Schematic illustration for the synthesis of Ce@SiO_2_ NGs and COx@Ce@SiO_2_ NGs, with their applications to colorimetrically detect H_2_O_2_ and choline.

**Figure 2:**
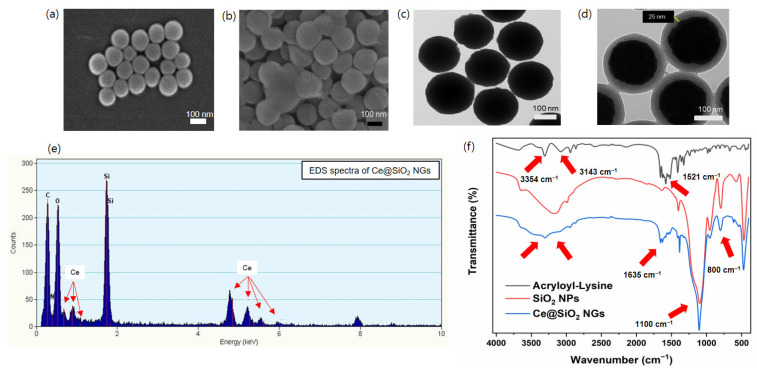
SEM images of (**a**) SiO_2_ NPs and (**b**) Ce@SiO_2_ NGs. TEM images of (**c**) SiO_2_ NPs and (**d**) Ce@SiO_2_ NGs. (**e**) EDS and (**f**) FT-IR spectra of Ce@SiO_2_ NGs.

Peroxidase-like Ce@SiO_2_ NGs, synthesized via a biocatalytic ATRP reaction, were used to construct cascade nanozyme COx@Ce@SiO_2_ NGs by entrapping COx for the convenient colorimetric detection of choline (Figure 1). The morphological characteristics of the Ce@SiO_2_ NGs were analyzed using SEM, which revealed a notable change in the dispersion of the SiO_2_ NPs after the polymer brush incorporation (Figure 2a,b). The bare SiO_2_ NPs were highly dispersed, while the Ce@SiO_2_ NGs displayed marginal aggregation, suggesting successful coating with the polymer brushes. The TEM images also showed a uniform spherical morphology with a diameter of approximately 160 nm of the bare SiO_2_ NPs (Figure 2c). After the surface modification, the Ce@SiO_2_ NGs exhibited an enlarged particle size due to the formation of outer polymeric brushes, with ~25 nm thickness, on the SiO_2_ core (Figure 2d). The EDS analysis validated the presence of C, O, N, Si, and Ce elements within the Ce@SiO_2_ NGs (Figure 2e), confirming the successful incorporation of the Ce ions in the nanogel matrix. The FT-IR spectroscopy further confirmed the successful synthesis of the Ce@SiO_2_ NGs (Figure 2f). The presence of Si-O-Si linkages was evident from the strong absorption peaks observed between 800 and 1100 cm^−1^, one of the characteristics of silica networks. Additional peaks at 1521 cm^−1^, 1635 cm^−1^, 3143 cm^−1^, and 3354 cm^−1^ were attributed to the stretching vibrations of C=O, C-H, N-H, and O-H, respectively, indicating the presence of the functional groups associated with the polymeric network and Ce coordination [26,27,28]. In addition, the XPS full spectra for the Ce@SiO_2_ NGs revealed the presence of C, O, N, Si, and Ce elements, which is consistent with the EDS analysis (Figure S1). The high-resolution XPS spectra presented special peaks of Si 2p, N 1s (N–H, C–N), and Ce 3d, which were observed at 103.1, 399.2, and 401.2, respectively [33,34]. The Ce@SiO_2_ NGs showed an extremely hydrophilic property, demonstrated by the contact angle of ~16°, which was 3-fold smaller than that of the SiO_2_ NPs (Figure S2). The enhanced hydrophilicity of the Ce@SiO_2_ NGs is presumed by the carboxyl and hydroxyl groups on their surface, which was confirmed by the FT-IR analysis. These spectral features collectively confirm the successful formation of Ce-coordinated nanogels with the intended structural and functional characteristics.

### 3.2. Investigation of Peroxidase-like Activity of Ce@SiO_2_ NGs

**Figure 3:**
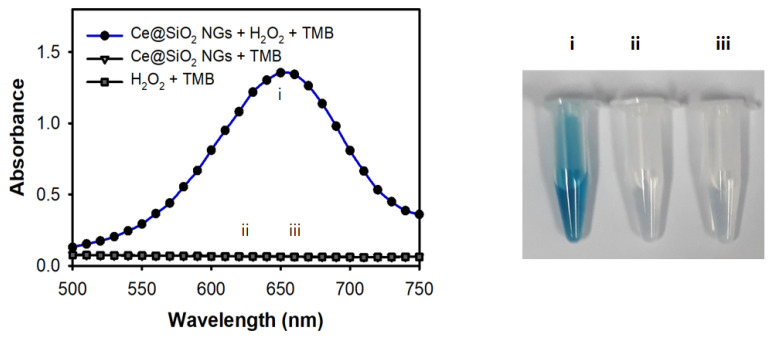
Peroxidase-like activity of Ce@SiO_2_ NGs through the oxidation of TMB in the presence of H_2_O_2_. The assay was performed in NaAc buffer (0.1 M, pH 4) containing Ce@SiO_2_ NGs (0.1 mg/mL) and TMB (1 mM) for 5 min incubation at RT. Sample specifications: i: Ce@SiO_2_ NGs + H_2_O_2_ + TMB, ii: Ce@SiO_2_ NGs + TMB, and iii: H_2_O_2_ + TMB.

The peroxidase-like activity of the Ce@SiO_2_ NGs was evaluated by performing the peroxidase-mediated oxidation of TMB in the presence of H_2_O_2_. First, the influences of pH and temperature on the catalytic activity were explored. The results showed that the Ce@SiO₂ NGs exhibited their maximal activity at pH 4.0 and 37 °C (Figure S3a,b); however, to facilitate practical utilizations, RT was used in further assays where the activity was more than 80% of that observed at 37 °C. During only a 5 min reaction, the Ce@SiO_2_ NGs successfully catalyzed the oxidation of TMB, producing a strong blue color signal in the presence of H_2_O_2_, but showed no activity without H_2_O_2_ (Figure 3). In addition, the incorporation of Ce ions within the SiO_2_ NPs played a key role in mimicking the peroxidase activity when SiO_2_-Pol(Lys) NPs, as a control, did not oxidize TMB in the presence of H_2_O_2_ (Figure S4). Moreover, the Ce@SiO_2_ NGs exhibited remarkable stability across broad pH and temperature ranges, contrasting with HRP, which rapidly lost activity under acidic conditions and elevated temperatures (Figure S3c,d). During the catalytic action of the Ce@SiO_2_ NGs in the presence of H_2_O_2_, the production of hydroxyl radicals was confirmed by employing a terephthalic acid (TA) probe, indicating that TMB can be oxidized with the hydroxyl radicals, similar to HRP-mediated catalysis (Figure S5). These results proved that trapped Ce ions within SiO_2_ NPs using a biocatalytic ATRP reaction notably enhanced the peroxidase-like activity and stability, which may serve as a feasible alternative to HRP in versatile applications.

To fully elucidate the peroxidase activity of the Ce@SiO_2_ NGs, their steady-state kinetic parameters were determined. The results showed that the Ce@SiO_2_ NGs followed Michaelis–Menten kinetics for both TMB and H_2_O_2_ (Figure S6a,b), and the kinetic parameters, *K_m_* and *V_max_*, were determined via Lineweaver–Burk plots (Figure S6c,d). Importantly, the *K_m_* values of the Ce@SiO_2_ NGs for TMB and H_2_O_2_ were found to be 0.57 and 1.79 mM, respectively, demonstrating a strong affinity when compared with other conventional nanozymes and HRP (Table S1). The enhanced affinity may be due to the polymeric brushes located outside the nanogels, which provide dynamic microenvironments around coordinated Ce ions, providing improved hydrophilicity and facilitating substrate transfer to the active site of the Ce@SiO_2_ NGs. These kinetic parameters demonstrate the high catalytic efficiency of Ce@SiO_2_ NGs, highlighting their potential as a robust alternative to natural HRP for the colorimetric detection of H_2_O_2_ and biomarkers in various analytical applications.

### 3.3. Quantitative Detection of H_2_O_2_ Using Ce@SiO_2_ NGs

**Figure 4:**
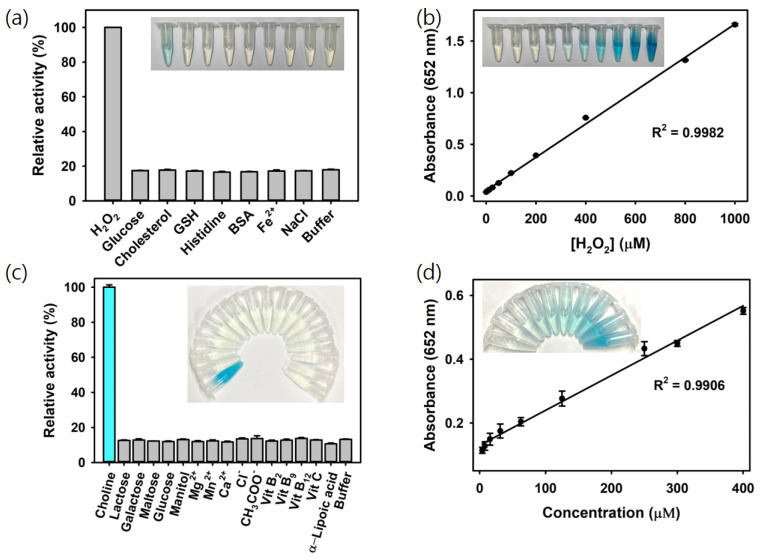
(**a**) Selectivity and (**b**) linear calibration plots to show sensitivity for the colorimetric detection of H_2_O_2_ using Ce@SiO_2_ NGs. (**c**) Selectivity and (**d**) linear calibration plots to show sensitivity for the colorimetric detection of choline using COx@SiO_2_ NGs.

The oxidation of TMB via H_2_O_2_ catalyzed by Ce@SiO_2_ NGs produces a vivid blue color signal, directly proportional to the concentration of H_2_O_2_. Leveraging this reaction, a colorimetric strategy for H_2_O_2_ detection using Ce@SiO_2_ NGs was developed. As a result, H_2_O_2_ was specifically detected during a 5 min reaction, whereas no significant color change was observed for the negative control samples, such as glucose, cholesterol, glutathione (GSH), histidine, bovine serum albumin (BSA), Fe^2+^, and NaCl, at the same concentration with H_2_O_2_ (5 mM), confirming the excellent specificity of the assay system towards the target H_2_O_2_ (Figure 4a). The limit of detection (LOD) value was calculated using the formula LOD = 3 × δ/slope, where δ is the standard deviation of the blank and slope is the slope of the calibration curve. Through the analysis of the dose–response curve, the LOD for H_2_O_2_ was determined to be as low as 1.3 μM, with a linear range from 5 to 1000 μM (Figure 4b). The LOD and linear range values are among the most sensitive reported for colorimetric H_2_O_2_ detection, making this system highly advantageous for quantifying H_2_O_2_ and other biomarkers when combined with their respective oxidase enzymes (Table 1).

### 3.4. Quantitative Detection of Choline Using COx@Ce@SiO_2_ NGs

H_2_O_2_ is a byproduct of the natural oxidase-mediated catalysis of biomarkers, and the COx@SiO_2_ NG-based system exhibited high selectivity and sensitivity in detecting H_2_O_2_. Based on these observations, we developed a detection system for choline, an important neurotransmitter and biomarker for monitoring various diseases and nutritional status, by incorporating COx into Ce@SiO_2_ NGs to construct COx@Ce@SiO_2_. In the presence of choline, the entrapped COx catalyzes the oxidation of choline to produce H_2_O_2_, which subsequently activates the peroxidase-mimicking Ce@SiO_2_ to produce a visible blue color signal from the oxidation of TMB. To optimize the preparation conditions of COx@Ce@SiO_2_, we investigated the effects of the COx concentration on its loading and choline detecting activity (Figure S7). The porous nature of the Ce@SiO_2_ NGs is expected to be advantageous to entrap COx, and, at 0.1 mg/mL COx, the maximum loading (~28 wt%) and activity were achieved, indicating that this concentration is optimal for the formation of COx@Ce@SiO_2_ NGs.

Using the optimized conditions with the COx@Ce@SiO_2_ NGs, choline was specifically detected by the intense blue color produced, while no noticeable color change occurred in the negative controls, including common interferes in milk such as lactose, galactose, maltose, glucose, manitol, Mg^2+^, Mn^2+^, Ca^2+^, Cl^−^, CH_3_COO^−^, vitamin B_2_, B_9_, B_12_, C, and α-lipoic acid, demonstrating the high specificity of the COx@Ce@SiO_2_ NG-based system towards choline (Figure 4c). The linear calibration plots showed the LOD as low as 2 μM, with a linear range from 4 to 400 μM (Figure 4d). These LOD and linear range values rank among the most sensitive reported for colorimetric choline detection (Table 2). These results proved that the developed biosensor could serve as a simple and highly sensitive approach for H_2_O_2_ and choline analysis without using expensive and specialized instruments or complex labeling procedures, making it a valuable tool for choline and food safety analysis in dairy products and infant nutrition.

The storage stabilities of the COx@Ce@SiO_2_ NGs and a free enzyme system comprising free HRP and free COx were evaluated under different temperature conditions (4 °C, RT, and 37 °C). The results showed the improved stability of the COx@Ce@SiO_2_ NGs under different temperature conditions (Figure S8), which may be attributed to the protective polymeric brushes of the nanogel. When nanogels were employed, the choline detecting activity was maintained over 80% during 6 days at RT, whereas the free system lost over 40% of its initial activity. When stored at 37 °C, there was a marginal decrease in the COx@Ce@SiO_2_ NGs, probably due to the temperature-dependent denaturation and release of the immobilized COx.

### 3.5. Choline and H_2_O_2_ Detection in Milk and Infant Formula Samples

Choline is predominantly found in infant formula and fresh milk, making its accurate detection crucial for ensuring nutritional quality. To assess the practical applicability of the COx@Ce@SiO_2_ NG-based assay, commercially available milk and infant formula were used to prepare the spiked sample for analysis. Initially, the original concentrations of choline in the fresh samples were determined using commercial assay kits, followed by the addition of specific amounts of choline to prepare the spiked samples. As a result, the choline levels in the spiked samples were measured with high precision and accuracy, with CVs in the range of 0.3 to 4.1% and recoveries of 97.5 to 105.3%. Additionally, the NGs designed for H_2_O_2_ detection demonstrated great analytical performance, with recoveries for the spiked H_2_O_2_ in the milk and infant formula samples ranging from 97.5 to 103.7% and CVs between 1.2 and 4.0%, underscoring the method’s reliability and reproducibility (Table 3). These results highlight the capability of using the COx@Ce@SiO_2_ NG-based assay to effectively analyze real food matrices, such as dairy products, ensuring the safety and nutritional adequacy of infant food sources.

## 4. Conclusions

We demonstrated that Ce@SiO_2_ NGs and COx@Ce@SiO_2_ NGs present a significant advancement in the colorimetric detection of H_2_O_2_ and choline, respectively. The ATRP-based synthesis of Ce@SiO_2_ NGs resulted in well-defined polymer-coated nanoparticles with excellent peroxidase-like activity. High selectivity and sensitivity towards target H_2_O_2_ and choline were achieved, underscoring their applicability in real-world scenarios. Validation through assays using commercial milk and infant formula confirmed the reliability and accuracy in practical applications. This research highlights the potential of ATRP-based nanozymes not only for enhancing food safety analysis in dairy products but also for broader biosensing applications, paving the way for future nanozymatic innovations in this field.

## Figures and Tables

**Table 1:** Comparison of analytical performances of various nanozymes for H_2_O_2_ detection.

Sample	Linear Range (μM)	LOD (μM)	References
Poly(ANI-*co*-AA) composite film	25–200	35.6	[35]
Fe_3_O_4_ QDs	10–400	4.5	[36]
CS@GSH-CuNCs	20–200	6.7	[37]
Au/Co_3_O_4_-CeOx NCs	10–100	5.29	[38]
Fe–Ag_2_S	10–150	7.82	[39]
Ag@TPE-SiO_2_ NPs	5–160	2.1	[40]
Co/CeO_2_	3.33–100	3.33	[41]
Ce@SiO_2_ NGs	5–1000	1.3	This work

**Table 2:** Comparison of analytical performances of various nanozymes for choline detection.

Sample	Linear Range (μM)	LOD (μM)	References
Au/HZIF-8@TCPP(Fe)	50–2000	50	[42]
ChO*_x_*@MOF	6–300	2	[43]
Mn/ZIF-90	5−50 and 50−1000	5.6	[44]
CS@GSH-CuNCs	20–150	6.5	[37]
Ce@SiO_2_ NGs	4–400	2	This work

**Table 3:** Detection precision of COx@Ce@SiO_2_ NGs and Ce@SiO_2_ NGs for choline and H_2_O_2_ quantification, respectively, in spiked milk and infant formula samples.

		Original Amount (µM)	Spiked Level (µM)	Measured (µM)	Recovery (%) (*n* = 3)	CV (%)
Choline	Milk #1	7.9	50	57.0	98.4	3.2
			100	111.0	102.8	3.6
			200	209.8	100.9	0.3
	Milk #2	6.6	50	57.2	101.1	2.3
			100	103.9	97.5	1.3
			200	204.7	99.1	1.0
	Infant formula #1	8.2	50	59.2	101.8	3.8
			100	111.4	103.0	1.1
			200	214.0	102.8	2.1
	Infant formula #2	8.1	50	57.6	99.2	3.6
			100	109.3	101.1	4.1
			200	219.0	105.3	2.4
H_2_O_2_	Milk #1	0	25	25.9	103.7	1.8
			50	50.8	101.5	1.2
			100	99.5	99.5	2.7
	Milk #2	0	25	25.24	101.0	2.5
			50	49.4	98.8	3.6
			100	101.8	101.8	2.7
	Infant formula #1	0	25	25.7	103.0	2.4
			50	50.4	100.8	4.0
			100	97.5	97.5	2.7
	Infant formula #2	0	25	25.1	100.3	2.5
			50	51.3	102.6	3.1
			100	100.6	100.6	2.6

## Data Availability

Data are contained within the article and Supplementary Materials.

## Supplementary Materials

The following supporting information can be downloaded at https://www.mdpi.com/article/10.3390/bios14120563/s1, Figure S1: XPS spectra of (a) Ce@SiO_2_ NGs and their high-resolution XPS spectra of (b) Si 2p, (c) N 1s, and (d) Ce 3d; Figure S2: Contact angle of (a) SiO_2_ NPs and (b) Ce@SiO_2_ NGs; Figure S3: Effects of (a) pH and (b) temperature on the peroxidase-like activity of Ce@SiO_2_ NGs. Catalytic stabilities of Ce@SiO_2_ NGs and HRP in ranges of (c) pH and (d) temperature; Figure S4: Peroxidase-like activity of (i) Ce@SiO_2_ NGs, (ii) SiO_2_-Pol(Lys) NPs, and (iii) 10 mM H_2_O_2_; Figure S5: Demonstration of hydroxyl radicals produced during the catalytic action of Ce@SiO_2_ NGs and HRP in the presence of H_2_O_2_. In the assays, 10 mM H_2_O_2_ and 0.625 mM TA were employed; Figure S6: Steady-state kinetic assays of Ce@SiO_2_ NGs for (a) TMB and (b) H_2_O_2_ and their corresponding double reciprocal (Lineweaver–Burk) plots of activity; Figure S7: (a) COx loading percentage within Ce@SiO_2_ NGs (1mg/mL) and (b) relative activity to detect choline prepared at various initial COx concentrations; Figure S8: Storage stability of (a) free enzyme system comprising free HRP and free COx and (b) COx@Ce@SiO_2_ NGs under different temperature conditions; Table S1: Comparison of peroxidase-like kinetic parameters of Ce@SiO_2_ NGs and other catalysts. References [45,46] are cited in the Supplementary Materials.
