# Supplementary material for: Choline Oxidase-Incorporated ATRP-Based Cerium Nanogels as Nanozymes for Colorimetric Detection of Hydrogen Peroxide and Choline
Source: Biosensors (Basel). 2024 Nov 21;14(12):563. doi: 10.3390/bios14120563 (PMC11675018; doi:10.3390/bios14120563)
Supplement: Supplementary file 1 [file biosensors-14-00563-s001.zip › biosensors-3300274-supplementary.pdf]

biosensors

Supplementary Materials

Choline Oxidase-Incorporated ATRP-Based Cerium-Nanogels as Nanozymes for Colorimetric Detection of Hydrogen Peroxide and Choline

Trung Hieu Vu [1], Byung Jo Yu [2,*] and Moon Il Kim [1,*]

1 Department of BioNano Technology, Gachon University, 1342 Seongnamdae-ro, Sujeong-gu, Seongnam, Gyeonggi 13120, Republic of Korea; hieu.vutrung24596@gmail.com (T.H.V)

2 Low-Carbon Transition R&D Department, Research Institute of Sustainable Development Technology, Korea Institute of Industrial Technology (KITECH), Cheonan 31056, Republic of Korea

* Correspondence: moonil@gachon.ac.kr (M.I.K.); bjyu@kitech.re.kr (B.J.Y.)

Synthesis of N-acryloyl-L-lysine

L-lysine hydrochloride (45 mmol), $CuSO_4.5H_2O$ (25 mmol), NaOH (90 mmol), Na_2CO_3 (55 mmol) were dissolved in 40 mL of H_2O, followed by the dropwise addition of acryloyl chloride (55 mmol) at 0°C, forming a blue precipitate. The resulting blue precipitate was filtered, washed sequentially with H_2O, ethanol, and petroleum ether, and then dried. The dried solid was then suspended in 200 mL solution (H_2O and chloroform = 1:1), followed by the addition of excess amount of 8-hydroxyquinoline. After 1 h vigorous stirring, the green solid was obtained, filtered, and washed with H_2O. The filtrate was separated into two layers. The aqueous layer was washed with dichloromethane, and the desired white solid was finally obtained by freeze-drying.

Synthesis of SiO_2 NPs

SiO_2 NPs were synthesized via modified Stöber process. Tetraethyl orthosilicate (2 mL) was dropped into 100 mL ethanol (98%) containing 7.5 mL ammonia solution, followed by stirring for 2 h at 65°C. SiO_2 NPs were collected by centrifugation at 10,000 *g* for 5 min and washed several times with absolute ethanol and redispered in ethanol. Subsequently, 200 μL APTES was added and the mixture was then stirred at 70°C for 24 h. the aminated SiO_2 NPs (SiO_2–NH_2 NPs) were collected by centrifugation at 10,000 *g* for 5 min, and washed with absolute ethanol. After that, 100 mg SiO_2–NH_2 NPs and 10 mg NHS-Bib were added into 200 mL of PBS (pH 7.4) and stirred overnight, to obtain bromine-modified SiO_2 NPs (SiO_2-Br NPs) collected by centrifuging and washing with absolute ethanol several times.

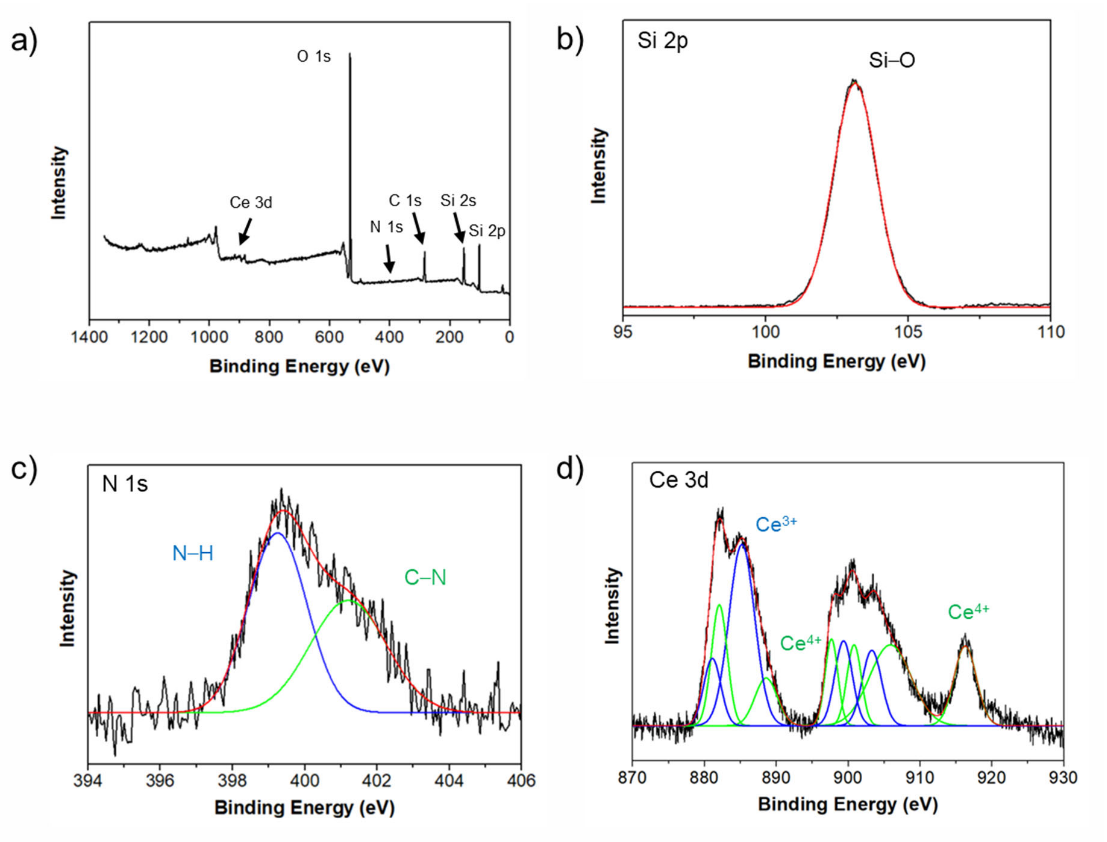

Figure S1. XPS spectra of a) Ce@SiO_2 NGs and their high-resolution XPS spectra of b) Si 2p, c) N 1s, and d) Ce 3d.

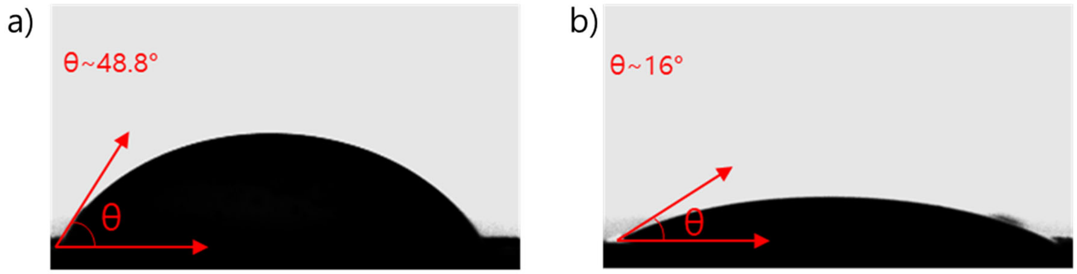

Figure S2. Contact angle of (a) SiO_2 NPs and (b) Ce@SiO_2 NGs.

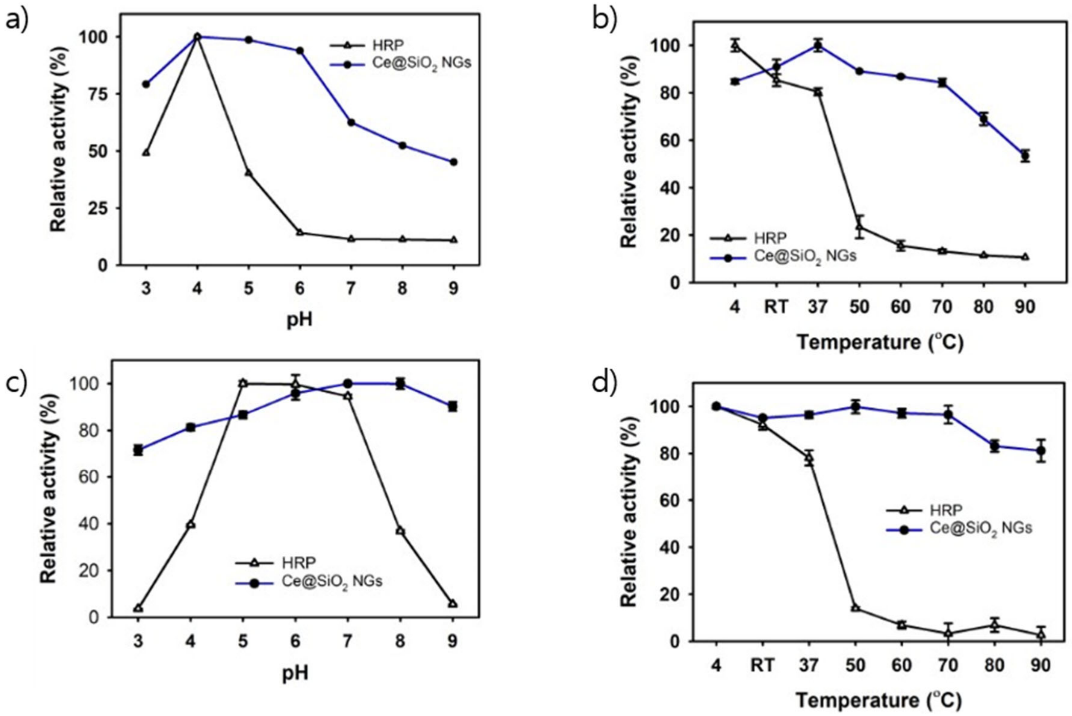

Figure S3. Effects of a) pH and b) temperature on the peroxidase-like activity of Ce@SiO_2 NGs. Catalytic stabilities of Ce@SiO_2 NGs and HRP in ranges of c) pH and d) temperature.

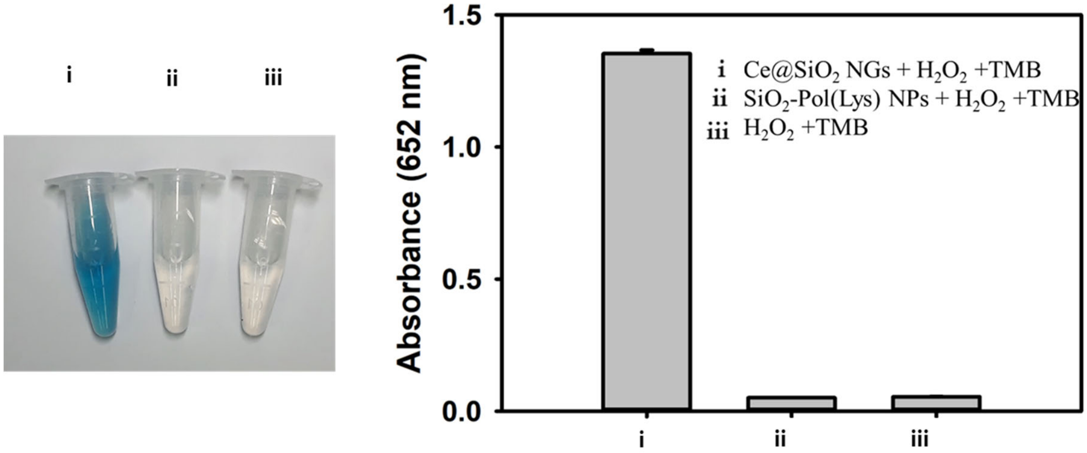

Figure S4. Peroxidase-like activity of (i) Ce@SiO_2 NGs, (ii) SiO_2-Pol(Lys) NPs, and (iii) 10 mM H_2O_2.

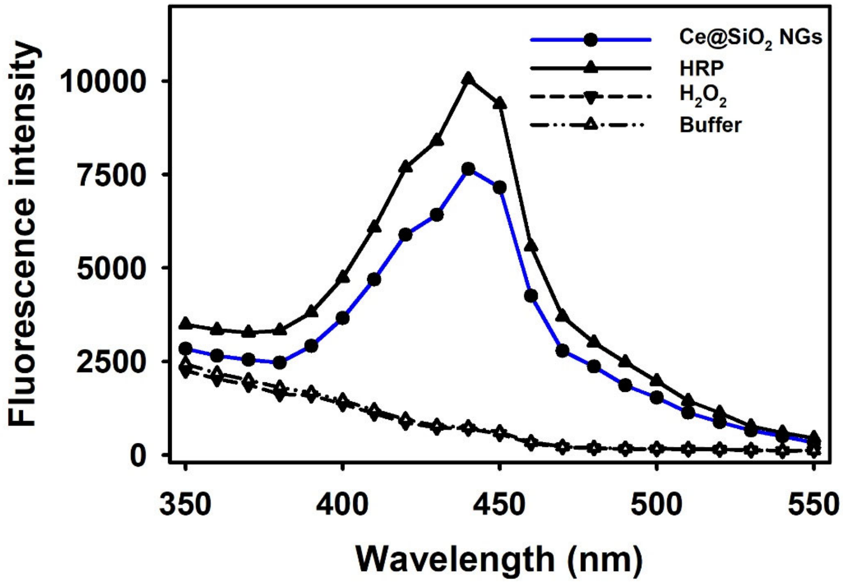

Figure S5. Demonstration of hydroxyl radicals produced during the catalytic action of Ce@SiO_2 NGs and HRP in the presence of H_2O_2. In the assays, 10 mM H_2O_2 and 0.625 mM TA were employed.

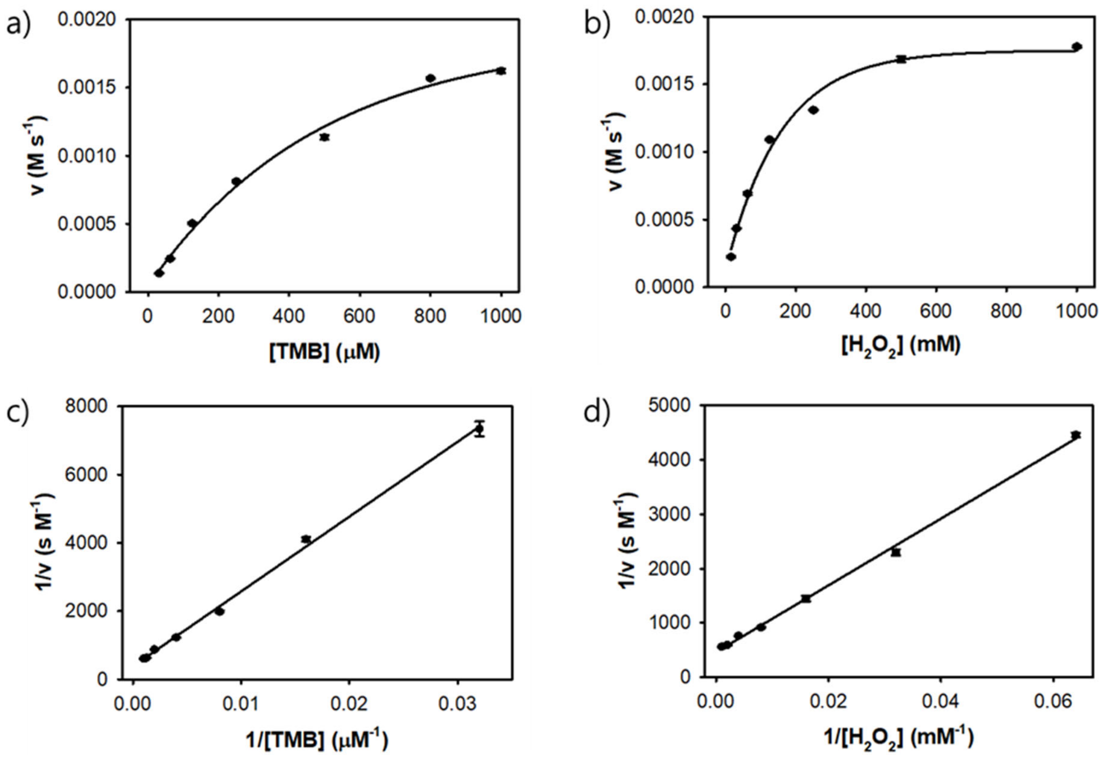

Figure S6. Steady-state kinetic assays of Ce@SiO_2 NGs for a) TMB and b) H_2O_2, and their corresponding double reciprocal (Lineweaver-Burk) plots of activity.

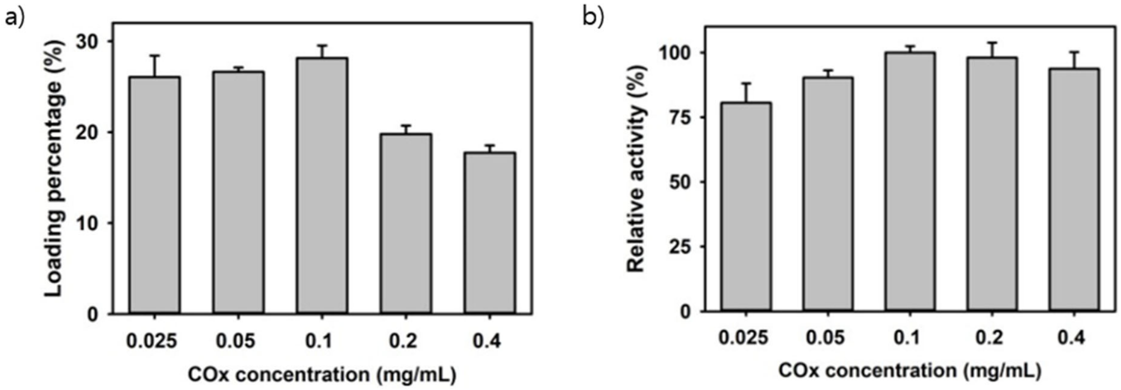

Figure S7. a) COx loading percentage within Ce@SiO_2 NGs (1 mg/mL) and b) relative activity to detect choline prepared at various initial COx concentrations.

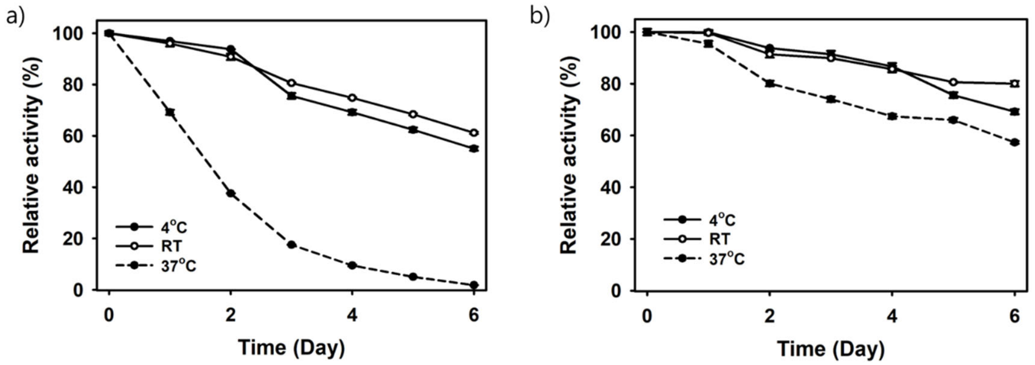

Figure S8. Storage stability of (a) free enzymes system comprising free HRP and free COx and (b) COx@Ce@SiO_2 NGs under different temperature conditions.

Table S1. Comparison of peroxidase-like kinetic parameters of Ce@SiO_2 NGs and other catalysts.

Sample	Substrate	K_m (mM)	V_{max} (μM s^{-1})	References
Co_3O_4/CeO_2	TMB	0.36	0.167	[45]
	H_2O_2	132.21	0.043	
CeO_2	TMB	0.42	0.137	
	H_2O_2	138.31	0.040	
MNP	TMB	0.098	0.034	[46]
	H_2O_2	154	0.0978	
HRP	TMB	0.43	0.01	
	H_2O_2	3.70	0.0870	
Ce@SiO_2 NGs	TMB	0.57	0.362	This work
	H_2O_2	1.79	0.119	

© 2024 by the authors. Submitted for possible open access publication under the terms and conditions of the Creative Commons Attribution (CC BY) license (https://creativecommons.org/licenses/by/4.0/).
